# Quantitative Hydrogen Chloride Detection in Combustion Environments Using Tunable Diode Laser Absorption Spectroscopy with Comprehensive Investigation of Hot Water Interference

**DOI:** 10.1177/00037028211060866

**Published:** 2022-01-04

**Authors:** Wubin Weng, Jim Larsson, Joakim Bood, Marcus Aldén, Zhongshan Li

**Affiliations:** Division of Combustion Physics, 5193 Lund University, Lund, Sweden

**Keywords:** Hydrogen chloride, tunable diode laser absorption spectroscopy, TDLAS, combustion–gasification environments, hot water line, high temperature, biomass, waste

## Abstract

Hydrogen chloride (HCl) monitoring during combustion/gasification of biomass fuels and municipal solid waste, such as polyvinyl chloride (PVC) and food residues, is demanded to avoid the adverse effect of HCl to furnace operation and to improve the quality of the gas products. Infrared tunable diode laser absorption spectroscopy (IR-TDLAS) is a feasible nonintrusive in-situ method for HCl measurements in harsh environments. In the present work, the measurement was performed using the R(3) line of the ν_2_ vibrational band of HCl at 5739.25 cm^–1^ (1742.4 nm). Water vapor is ubiquitous in combustion/gasification environments, and its spectral interference is one of the most common challenges for IR-TDLAS. Spectral analysis based on the current well-known databases was found to be insufficient to achieve an accurate measurement. The lack of accurate temperature-dependent water spectra can introduce thousands parts per million (ppm) HCl overestimation. For the first time, accurate spectroscopic data of temperature-dependent water spectra near 5739.3 cm^–1^ were obtained based on a systematic experimental investigation of the hot water lines in a well-controlled, hot flue gas with a temperature varying from 1100 to 1950 K. With the accurate knowledge of hot water interference, the HCl TDLAS system can achieve a detection limit of about 100 ppm⋅m at around 1500 K, and simultaneously the gas temperature can be derived. The technique was applied to measure the temporally resolved HCl release and local temperature over burning PVC particles in hot flue gas at 1790 K.

## Graphical abstract

**Graphical Abstract:**
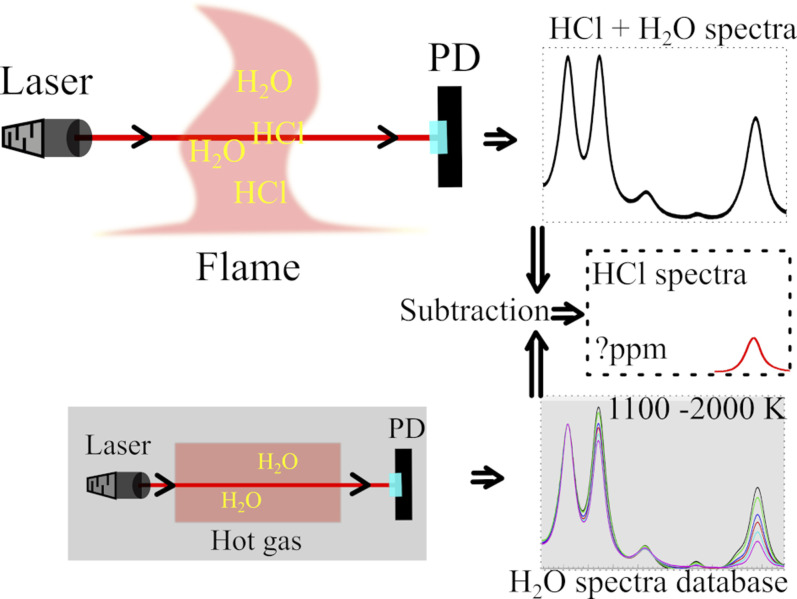

## Introduction

Thermochemical conversion processes, such as combustion and gasification, are common ways to process municipal solid waste and agriculture/forest residues for energy and material recycling. However, the high amount of chlorine in the feedstocks, such as polyvinyl chloride (PVC), food residues with sodium chloride (NaCl) and some agriculture residue with high potassium chloride content, can introduce severe problems to the operation of the combustion/gasification systems. Chloride salts (sodium chloride, potassium chloride, zinc chloride, etc.) induce corrosion and slagging to the surface of high-temperature heat exchangers due to their low melting point and high corrosion.^1^ Hydrogen chloride (HCl) in the flue gas or syngas product can cause dew point corrosion and catalyst poisoning. Thus, it is essential to fully understand the chlorine chemistry and the fate of chlorine in combustion/gasification environments. HCl is one of the dominant chlorine species in high-temperature thermochemical reaction processes.^2^ Accurate HCl concentration measurement in combustion/gasification environments is therefore essential.

**Figure 1.**
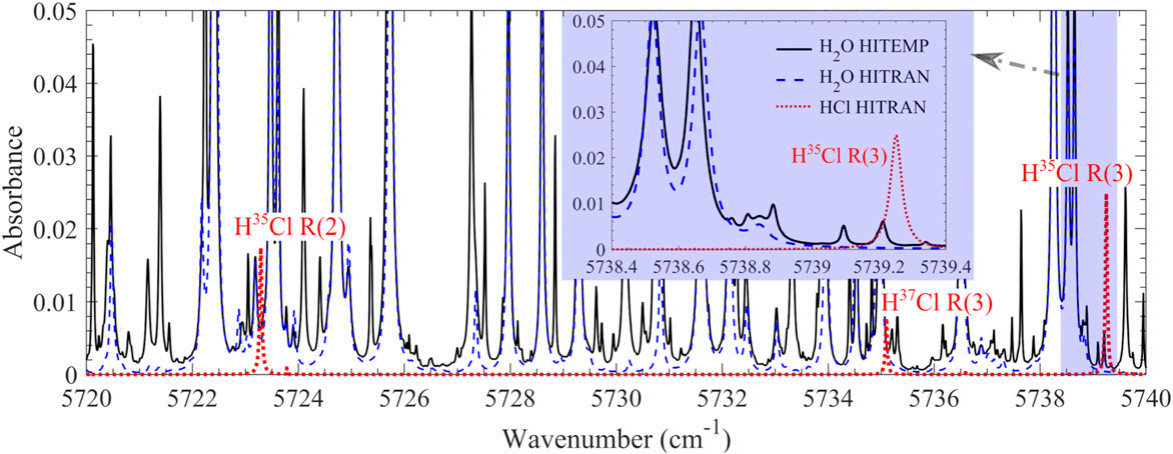
Calculated absorbance of 10% H_2_O and 1000 ppm HCl at 1600 K and atmospheric pressure with an optical path length of 1 m. The spectra data from HITRAN2016^11^ and HITEMP2010^12^ databases were adopted for spectral simulation. Inset: High resolution spectra at 5738.4–5739.4 cm^–1^.

Sampling is a conventional way to measure HCl concentration. However, the high reactivity of HCl, especially below the dew point, introduces substantial uncertainties in measurements. Different optical methods have been proposed to achieve in situ nonintrusive measurements. Using ro-vibrational bands, including the fundamental band ν_1_ (∼3.5 µm), the first overtone band ν_2_ (∼1.7 µm) and the second overtone band ν_3_ (∼1.2 µm), a variety of infrared (IR) spectroscopy techniques have been developed. Li et al.^3^ demonstrated the detection of HCl in the burnt region of a methane flame with chloroform (CHCl_3_) seeding using mid-infrared polarization spectroscopy (IRPS), where the rotational lines of the fundamental ro-vibrational band were probed. A detection limit less than 50 parts per million (ppm) was achieved. Sun et al.^4^ applied infrared degenerate four-wave mixing (IR-DFWM) to measure trace amounts of HCl in a nitrogen gas flow at room temperature, where the fundamental ro-vibrational band was also probed, and the detection sensitivity was estimated to be about 25 ppm. Tunable diode laser absorption spectroscopy (TDLAS) is another laser-based technique successfully developed for HCl detection. Compared with IRPS and IR-DFWM, TDLAS has multiple advantages. It can be calibration-free, relatively simple in experimental setup, economic, and robust, although its line-of-sight nature hinders spatially resolved measurement. Qu et al.^5^ and Nwaboh et al.^6^ developed HCl measurement using TDLAS probing the fundamental vibrational band. Linnerud et al.,^7^ Corsi et al.,^8^ Kim et al.,^9^ and Ortwein et al.^10^ used the overtone vibrational band of HCl, mostly the first overtone. The rotational lines of the first overtone at around 1.7 μm were regarded as a very suitable choice, whereas the second overtone has much weaker line intensity^8^ and the fundamental band requires more expensive equipment.^10^ For the HCl-TDLAS, using the first overtone at 1.7 μm, a detection limit down to 0.1 parts per million, or ppm (optical path length, 1 m), has been reported at room temperature.^7^ However, there are still challenges to apply this technique in combustion–gasification environments usually having a temperature over 1000 K. A high proportion of water vapor is usually contained in combustion–gasification environments, which generally contribute a significant spectral interference. It is of importance to select a suitable HCl absorption line to minimize water line interference. In the present work, the R(3) line of the *v*_2_ vibrational band at 5739.25 cm^–1^ with a line intensity (*S*) of 1.25·10^–20^ cm/molecule at 296 K was selected, where a low spectral interference from hot water lines was expected, based on the simulation using the HITRAN2016 database^11^ in the wavelength range between 5350 and 5850 cm^–1^ covering the whole HCl ν_2_ vibrational band. Part of the simulation result is shown in Fig. 1. Similar simulation using HITRAN database has been reported by Ortwein et al.,^10^ who have conducted HCl measurement in a gasification furnace up to 1800 K. However, when the simulation was conducted using the well-known HITEMP2010 database,^12^ significant amount of additional hot water lines emerged (Fig. I). The additional hot water lines near the HCl absorption line might introduce a significant measurement error. The error was estimated to be hundreds of ppm according to the simulation from the HITEMP2010 database (Fig. 1). The hot water absorption spectra have also been simulated by POKAZATEL,^13^ the most complete water line list to date. The results were similar to that by the HITEMP2010 database. To further clarify the interference on the measurement accuracy, a systematic investigation of the hot water lines in homogeneous high-temperature environments is needed. A laminar flame burner was employed to provide the hot gas environments covering temperatures from 1120 to 1950 K. The temperature-dependent, hot water line absorption spectra were measured, and a database is formed to compensate the interference from hot water in the measurements of HCl in combustion/gasification environments. As a demonstration of this approach, the time-resolved concentration of HCl released from burning PVC particles was measured.

## Experimental

**Figure 2.**
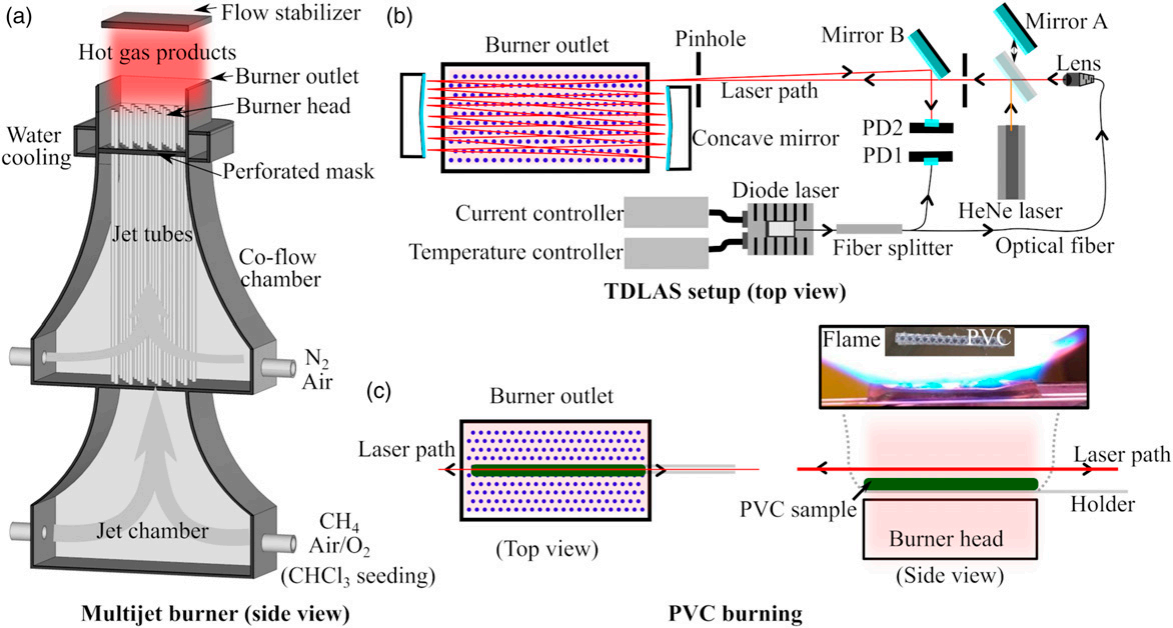
Schematic of the multi-jet burner (Reproduced from Weng et al.^18^ Copyright 2020 Elsevier.) (a), and the TDLAS setup for HCl measurement in the hot flue gas (b), and measurements above PVC sample (c). PD: photodiode.

A laminar flame multi-jet burner was used to provide the hot flue gases, which have a high degree of uniformity and good temperature adjustability, beneficial to the investigation of the HCl-TDLAS technique in combustion–gasification environments. The good temperature-uniformity is also one of the key prerequisites in obtaining accurate hot water spectra at a given temperature. A schematic of the multi-jet burner is shown in Fig. 2a, and its detail has been described by Weng et al.^14^ elsewhere. It consists of two chambers, that is, the jet chamber and the co-flow chamber. Premixed methane–air–oxygen was introduced into the jet chamber and evenly distributed into 181 jet tubes to generate premixed flames above each jet tube. Nitrogen and air were used as co-flow, which was introduced into the co-flow chamber and entered into the burner head after passing a perforated mask. After the mixing of the co-flow gas and the post-flame product of the premixed flames, homogeneous hot gas products were generated. The hot gas above the burner outlet (85 × 45 mm^2^) was used in the present investigation. The flame conditions used in the present work are listed in Table I. All gas flows were precisely controlled by mass flow controllers (Bronkhorst). The temperature of the hot gas 5 mm above the burner outlet was measured using two-line atomic fluorescence thermometry with indium atoms as reported by Borggren et al.^15^ The temperature was evenly distributed^14,15^ in a region of about 70 × 40 mm^2^, with a narrow temperature transition zone on the edge. This characteristic was essential for the investigation of the line-of-sight HCl-TDLAS technique. The concentration of the water vapor in the hot flue gas was estimated to be about 10% through chemical equilibrium calculation.

**Table I.** Summary of the flame conditions, where the temperature was measured at a location 5 mm above the burner outlet.

Flame Case	Gas flow rate (sl/min)					Fuel–O_2_ equivalence ratio ϕ	Gas product temperature (K)
	Jet flow			Co-flow			
	CH_4_	Air	O_2_	N_2_	Air		
F1	2.95	19.20	2.09	6.84	7.09	0.78	1950
F2	2.66	17.34	1.89	10.83	7.74	0.74	1750
F3	2.47	12.23	2.58	18.97	8.90	0.70	1550
F4	2.28	11.89	2.26	22.69	9.83	0.67	1390
F5	2.09	10.90	2.07	26.50	10.66	0.63	1260
F6	1.71	8.91	1.69	26.92	10.25	0.60	1120
F7	1.66	15.50	1.49	9.12	0.00	0.70	1540
F8	2.66	17.34	1.89	18.60	0.00	0.96	1790

Chloroform (CHCl_3_) vapor was seeded to generate hydrogen chloride (HCl) in the hot flue gas. Liquid chloroform was filled in a gas bubbler bottle kept at room temperature (23.5 °C). A nitrogen flow passed through the liquid chloroform and carried a known amount of chloroform vapor to the jet-flow chamber. The chloroform concentration was calculated based on its vapor pressure (0.241 bar at 23.5 °C^16^), and the concentration of HCl in the hot flue gas was calculated assuming all chloroform was decomposed in the fuel-lean premixed flames.^3,17^ The HCl concentration was changed from 104 to 832 ppm with a different flow rate of the carrier gas, from 4 to 32 mL.

As a demonstration measurement, the TDLAS technique in the present setup was applied to measure the release of HCl from burning PVC samples. As shown in Fig. 2c, 600 mg PVC particles were prepared and carried by a vessel held by two ceramic rods with a diameter of 1 mm. It was heated and burned in the center of the hot flue gas provided by flame F8 at 1790 K. A photo of the burning particles with a gas plume is shown in the inset of Fig. 2c.

The schematic of the TDLAS system is shown in Fig. 2b. A distributed feedback diode laser (Butterfly, Toptica) was used, which was controlled by a temperature control module (Thorlabs, TED 200 C) and a current control module (Thorlabs, LDC 201 C, 100 mA). The laser covers the wavelength range of 1741–1745 nm with operating temperatures of 5–45 °C. In the present work, the operating temperature was set to 30.4 °C. A continuum wave laser beam at 1742 nm was produced with a power of about 5 mW. The driving current was ramped between 70–90 mA to make the laser wavelength scan over 1 cm^–1^ at 100 Hz. The laser beam was split into two parts with a power ratio of 25:75 using a fiber splitter. The weak one was used as the reference beam monitored by an IR photodiode (InGaAs, detecting wavelength 0.9–2.6 μm, PDA10D2, Thorlabs), that is, PD1 in Fig. 2b and the strong one was used as the measurement beam. The measurement beam with a diameter of about 1 mm was collimated by a fiber collimation package containing an AR-coated (1.8–3.0 μm) spherical lens (*f* = 5.95 mm, *D* = 10 mm). Two silver-coated concave mirrors (*f* = 100 mm, *D* = 50 mm, Thorlabs) were adopted to achieve the multiple passes of the laser beam in the hot flue gas at 5 mm above the burner outlet. The total optical path length was estimated to be 1.43 m. After an alumina-coated flat mirror (Mirror B), the laser was finally guided to another IR photodiode (InGaAs, detecting wavelength 1.2–2.6 μm, PDA10D-EC, Thorlabs), that is, PD2. Meanwhile, a HeNe laser beam (632.8 nm) together with two pinholes and an alumina-coated flat mirror (Mirror A) on a mobile station was used to facilitate the optical alignment. Both the signals from the reference beam detected by PD1 and the measurement beam detected by PD2 were acquired by a data acquisition board (National Instrument). Thus, the number density of HCl, *N*, was measured using the TDLAS system based on the Beer–Lambert law

(1) $$Abs=-\ln\left(\frac{\left(I\left(\text{v}\right)-I_{\text{s}}\right)/Tr}{I_{0}\left(\text{v}\right)-I_{\text{s}0}}\right)=\;S\left(T\right)\cdot g\left(\text{v}-{\text{v}}_{0}\right)\cdot N\cdot L$$

where *Abs* is the absorbance of the absorbing species, and $I_{0}\left(\text{v}\right)$ and I(v) are the initial laser intensity and the intensity after the absorbing species as a function of laser frequency, *v*, respectively. The laser intensity after the absorption was collected by the measurement photodiode (PD2), while the initial laser intensity was obtained by the reference photodiode (PD1). To obtain the $I_{0}\left(\text{v}\right)$ and I(v) values that can be used in Eq. 1, correction based on the power ratio between the reference and measurement beam was conducted. *I*_s0_ and *I*_S_ is the wavelength-independent additive signal from PD1 and PD2, mainly contributed by the detector dark current, and the broadband thermal radiation from the flame. The detector dark current signal was obtained under the condition without running the laser, while the thermal radiation was negligible as the photodiodes were placed 1.5 m away from the flame and pinholes were used to block this emission. *Tr* is the transmission of the laser after soot or other particles in the flame. In most situations, such as in the clean hot flue gases provided by the laminar flames from the multi-jet burner, *Tr* was one. *S*(*T*) is the absorption line strength, *g*(v–v_0_) is the area normalized shape function, and *L* is the optical path length.

## Results and Discussion

**Figure 3.**
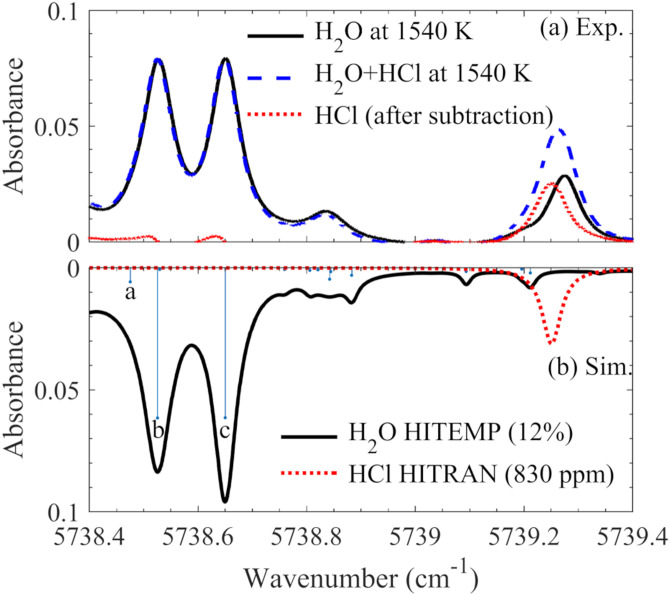
High resolution absorbance spectra of H_2_O and HCl measured in hot flue gas (*T* = 1540 K) provided by flame F7 (a), and the calculated ones of 12% H_2_O and 830 ppm HCl at 1540 K (b) using HITEMP2010 and HITRAN2016 database, respectively.

The absorption spectrum (5738.4 to 5739.4 cm^–1^) of the hot flue gas (*T* = 1540 K, *X*_H2O_ = 12%) provided by flame F7 is presented in Fig. 3a. Without HCl seeding, the absorption was attributed to H_2_O in the hot flue gas, while contributions from other dominant species, such as CO_2_, were excluded. Another absorption spectrum, indicated with blue dashed line in Fig. 3a, was obtained under the same conditions, but with about 830 ppm Cl addition, where chloroform was supposed to be fully converted into HCl. Between these two absorption spectra, a distinct difference was observed at the wavenumber around 5739.2 cm^–1^ (Fig. 3a, red dotted line), where the R(3) line of the *v*_2_ vibrational band of HCl locates. Thus, the absorption of HCl was the difference between these two spectra. Based on the HCl absorption, the HCl concentration can be calculated through the Beer–Lambert law. However, in this calculation process, a reference spectrum, that is, the spectrum of H_2_O, is needed for spectral subtraction. For most applications, the environment changes over time and it is hard to acquire the reference spectrum through the measurement directly. Predicting the H_2_O absorption near the HCl absorption line thus becomes critical to accurately measure HCl, especially considering that, as shown in Fig. 3a, the absorbance of 12% H_2_O is notable at 5739.2 cm^–1^.

Simulation of the water absorption is often performed with pre-knowledge of the temperature and H_2_O concentration. As shown in Fig. 3b, the absorbance spectrum of H_2_O was simulated using the HITEMP2010 database, where the temperature was set to be 1540 K and the concentration of H_2_O was 12%, same as the experimental condition. The major H_2_O transitions for these absorption lines are presented in Fig. 3b, which were all recognized as hot lines. The three strongest transitions are labeled a, b, and c in Fig. 3b. The spectral data of these transitions, from HITEMP2010, are presented in Table II, with transition wavenumber (*v*), line intensity (*S*) at 1540 K, lower-state energy (*E*) and the vibrational and rotational quantum numbers for the upper and lower states.

**Table II.** Spectra data of the strong H_2_O transitions (a, b, and c) in Fig. 3 obtained from HITEMP2010, including transition wavenumber (ν), line intensity (*S*) at 1540 K, lower-state energy (*E*) and the vibrational and rotational quantum numbers for the upper (′) and lower (″) states.

Tran.	ν (cm^–1^)	*S* (cm/molecule) [1540 K]	*E* (cm^–1^)	ν′	ν″	*J*′ *K*_a_′ *K*_c_′	*J*″ *K*_a_″ *K*_c_″
a	5738.476	8.23 × 10^–24^	5209.131	0 0 2	0 1 0	15 7 9	14 8 6
b	5738.526	8.79 × 10^–23^	3465.070	0 1 1	0 0 0	15 8 7	14 8 6
c	5738.650	8.78 × 10^–23^	4027.506	0 1 1	0 0 0	18 5 14	17 5 13

Compared to the experimentally measured absorbance spectrum, the simulation using the HITEMP2010 database can only provide a good prediction of the lines marked as a, b, and c. The weak water lines, especially the ones close to the HCl absorption line (5739.25 cm^–1^), were completely mis-predicted. In the simulation, two distinct weak lines close to 5739.2 cm^–1^ were predicted, but in fact, there was only one relatively strong line observed in our measurement, and the measured absorption line showed to be closer to the HCl absorption peak than the simulated ones. The larger overlap with the HCl absorption line implies that a thousand ppm error in the HCl measurement may be introduced if the H_2_O absorption was ignored. Therefore, systematical investigation of the hot water lines in this wavelength region at a variety of temperatures is crucial and inevitable for accurate HCl detection in combustion/gasification environments.

In the present work, three steps were adopted to tackle the aforementioned challenge. First, the temperature-dependent absorption spectra of the hot water lines between 5738.4 and 5739.4 cm^–1^ were obtained with a high spectral-resolution, and the measured spectra were made into a hot water line database. Second, for each measurement, the absorption spectrum of the two strongest water lines between 5738.4 and 5738.7 cm^–1^ was fitted by the H_2_O spectra in our database through an interception process, and the whole H_2_O absorption spectrum of each measurement can thus be predicted, especially the part near 5739.2 cm^–1^. Finally, the measured absorbance was subtracted by the predicted H_2_O absorbance. The remaining absorbance can be attributed to HCl absorption, and it can be fitted using the HITRAN database to determine the final HCl concentration.

**Figure 4.**
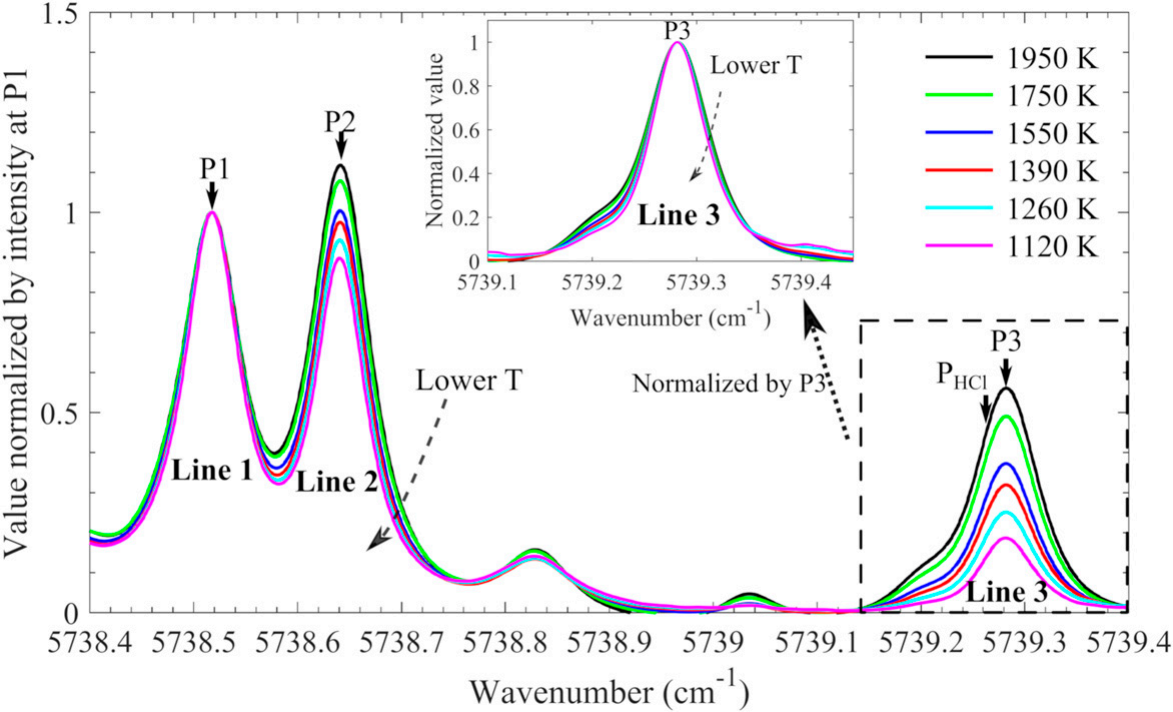
Measured H_2_O spectra normalized by the maximum absorbance of the hot water line, Line 1, as the temperature varied from 1120 K to 1950 K. Lines 1, 2, and 3 are the three strongest hot water lines between 5738.4 and 5738.8 cm^–1^, and P1, 2, and 3 are wavelengths of the peaks of the corresponding lines. The inset shows the spectra of the H_2_O line, Line 3, normalized by its own peak value.

Hot flue gases provided by flames can be used to mimic practical environments. In the present work, a laminar flame burner, that is, the multi-jet burner, was adopted to produce the hot flue gas environments with a temperature varied from 1120 to 1950 K (Table I). Temperature was the only variable as it is considered as the dominant factor influencing the profile of hot water absorption under atmospheric pressure combustion/gasification conditions. Because only hot lines were detected, the contribution from the water vapor in the cool ambient air was negligible. The absorption spectra of the hot water lines were obtained and are presented in Fig. 4. The three strongest hot water lines are labeled Line 1, Line 2, and Line 3, with corresponding peak wavenumbers, P1, P2, and P3, respectively. The absorbance spectra were normalized by the peak absorbance at P1 since Line 1 was found to be less sensitive to temperature variation according to the simulation. It can be seen, after the normalization, good overlap was obtained for Line 1, and the intensities of both Line 2 and Line 3 increase with temperature. Line 3 spectra were also normalized by its own peak value and are presented in the inset of Fig. 4. The normalized spectra cannot be perfectly unified, indicating that Line 3 may contain multiple transitions.

**Figure 5.**
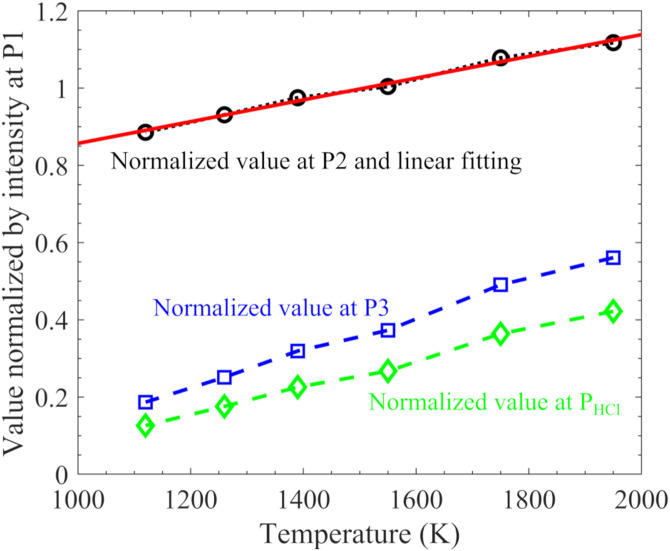
Normalized absorbance at position P2, P3, and P_HCl_ (as marked in Fig. 4), as a function of temperature, and corresponding linear fitting used for temperature estimation.

The normalized absorbance at wavelength P2, P3, and P_HCl_ (as marked in Fig. 4), as a function of temperature is presented in Fig. 5. Linear temperature dependence was observed at these three wavelengths, and Line 3 shows a larger variation than Line 2. The relationship between the normalized absorbance at P2, that is, *R*_P2_, and temperature, *T*, was linearly fitted (Fig. 5) and expressed by

(2) $$R_{P2}=0.0002817\times T+0.5753$$

Thus, in the present work, the temperature dependent *R*_P2_ was used for temperature estimation. The temperature information is not only an important parameter of combustion/gasification processes but also indispensable in the calculation of HCl concentration since the line intensity of HCl is temperature dependent.

The measured hot water absorption spectra (normalized by respective absorbance at P1) between 5738.4 and 5739.4 cm^–1^ at 1120, 1260, 1350, 1550, 1750, and 1950 K were packaged into a database, which is included in the Supplemental Material. In the HCl measurement, the absorption spectrum of Line 1 and Line 2 can be obtained directly from the measured spectrum since there was no contribution from HCl absorption at these wavenumbers. The absorbance at Line 2 (between the wavenumbers (λ) of 5738.6 and 5738.7 cm^–1^) was normalized by the one at P1, and the resulting value, $R_{\text{λ}}^{\text{L}2}$ , was used for the fitting of the whole water absorption spectrum through the interception of the normalized spectra in the database at the same wavenumbers, $R_{\text{λ}}^{T_{i}}$ , with minimizing deviation, $\underset{0\leq W\leq 1}{\min}\sum_{\text{λ}}\left|R_{\text{λ}}^{\text{L}2}-\left[W\cdot R_{\text{λ}}^{T_{i}}+\left(1-W\right)\cdot R_{\text{λ}}^{T_{i+1}}\right]\right|$ , where *W* is the weight and *T*_*i*=1:6_(1120, 1260, 1350, 1550, 1750, and 1950 K) is the temperature of the water spectra in the database. In the fitting calculation, the interception process is acceptable as the profile of the spectra was, in general, linearly varied with temperature as illustrated by Figs. 4 and 5.Through the water absorption spectral fitting, the whole water absorption spectrum, especially the Line 3 absorption part, was obtained, after multiplying $\left[W\cdot R^{T_{i}}+\left(1-W\right)\cdot R^{T_{i+1}}\right]$ with the absorbance at P1.

**Figure 6.**
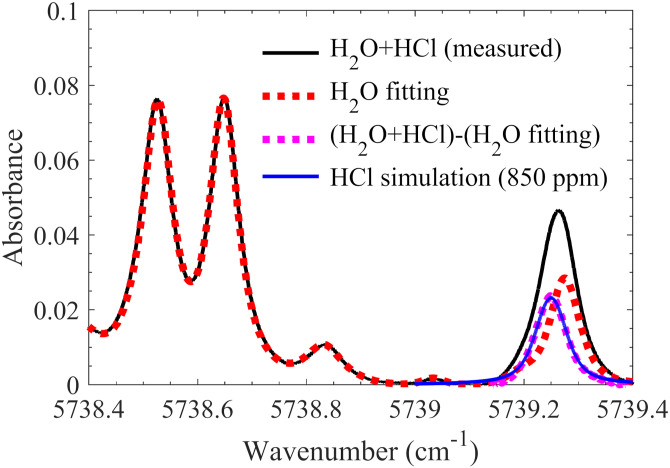
Absorbance spectrum obtained in the hot flue gas at 1540 K with 830 ppm Cl seeding (black line), the H_2_O absorbance spectrum fitted by the H_2_O spectra of our database at 1540 K (red dot line), the HCl absorption obtained after the subtraction of the water absorption (magenta dot line) and the corresponding fitting of 850 ppm HCl using HITRAN2016 database (blue line).

Figure 6 presents a typical absorbance spectrum obtained in the hot flue gas at 1540 K with 830 ppm Cl seeding (see black line in Fig. 6). After the fitting using the H_2_O spectra in the database, the whole H_2_O absorbance spectrum was obtained (see red dotted line in Fig. 6). The H_2_O absorbance was used to subtract the raw absorbance containing both HCl and H_2_O absorption. The remaining part was regarded as the measured HCl absorbance (see magenta dotted line in Fig. 6). Then, the measured HCl absorption spectrum was fitted with the line strength of HCl from the HITRAN2016 database and a Voigt line shape (see blue line in Fig. 6). In the curve fitting, a temperature of 1540 K was used and the HCl concentration was calculated to be 850 ppm with an uncertainty of ±50 ppm, which agrees well with the amount of Cl (830 ppm) seeded into the hot flue gas.

**Figure 7.**
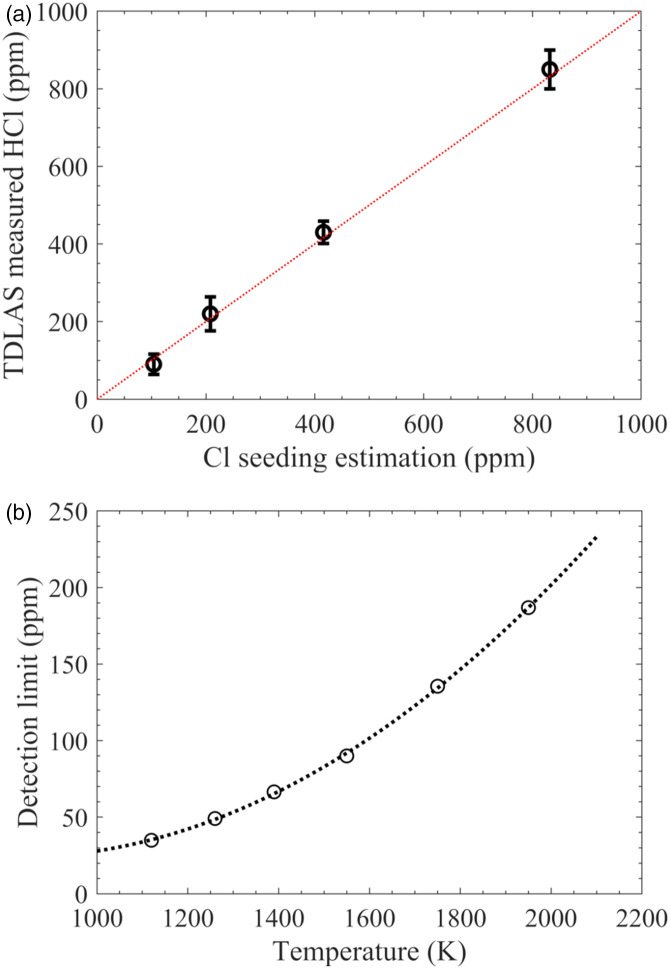
Measured HCl concentration using the present TDLAS system versus the amount of Cl seeded into the hot flue gas at 1540 K (a), and detection limit of the present TDLAS system as a function of temperature (b).

Through the addition of different amount of CHCl_3_ to flame F7, hot flue gas with different HCl concentration, that is, 104, 208, 416, and 832 ppm, was prepared. Correspondingly, 90, 220, 430, and 850 ppm HCl were measured, and the uncertainty was estimated to be around ±30 ppm, as shown in Fig. 7a, which was obtained based on the error in the HCl absorbance curve-fitting process. Thus, the detection limit was estimated to be around 100 ppm at a temperature around 1540 K. It is concluded that the detection limit is temperature dependent. Lower temperature leads to a higher HCl absorption intensity and a lower hot water line (Line 3) absorbance, which together results in a higher measurement sensitivity. This explains why in the previous studies at room temperature, the detection limit can reach down to 0.1 ppm.^7^ The estimated detection limit with an optical path length of 1.43 m at temperatures between 1000 and 2000 K is shown in Fig. 7b.

**Figure 8.**
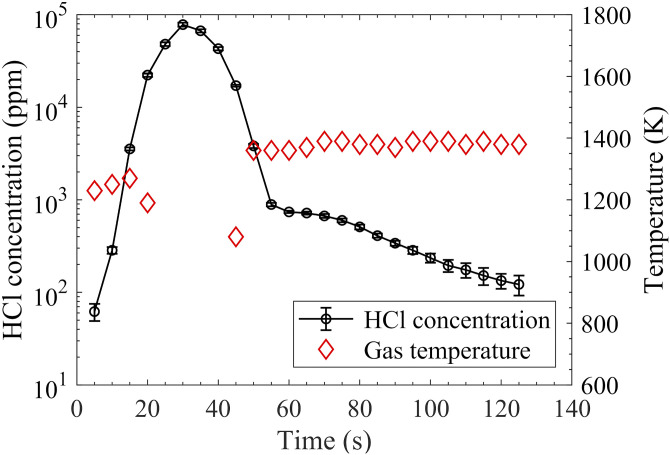
Concentration of HCl in the plume 5 mm above burning PVC particles in hot flue gas at 1790 K provided by flame F8 and the local temperature derived based on Eq. 2.

The TDLAS technique was also applied to measure the concentration of HCl in the gas plume above burning PVC particles. The PVC particles were prepared and filled in a vessel held by two ceramic rods in a hot flue gas at 1790 K provided by flame F8. The results are presented in Fig. 8. According to the HCl release process, the burning process can be divided into two stages, that is, a pyrolysis-burning stage and a char stage. At the first stage, the HCl concentration increased from 62 ± 13 ppm at a residence time of 5 s to its maximum, 78200 ± 3800 ppm at 30 s, and decreased to 890 ± 39 ppm at a residence time of 55 s. After that, only small amount of char was left and continuing HCl release was detected with a mild decrease to 122 ± 30 ppm at 130 s. Meanwhile, the temperature at the center of the plume was also obtained based on the correlation between temperature and the normalized absorbance at wavelength P2, that is, *R*_P2_, (Eq. 2). The temperature in the plume was about 300–400 K lower than the one of the ambient hot flue gases at 1790 K, which is consistent with the temperature measurement conducted in the volatile plume of burning biomass particles.^19–21^ At the beginning, the temperature was 1230 K. The enhanced volatile gas release decreased the temperature to around 1000 K. When the char stage started, the temperature of the gas increased back to around 1400 K. Under the conditions with a high amount of HCl release, the strong HCl absorption dominated the hot water lines, Line 1 and Line 2, and the temperature cannot be accurately obtained. However, the water line absorption interference from Line 3 on the HCl measurement was negligible as the HCl absorption is over 100 times higher than the H_2_O Line 3. In the present work, a temperature of 1000 K was used to determine the line intensity of HCl under the conditions with a strong HCl release.

## Conclusion

The work demonstrated the measurement of HCl in combustion/gasification environments using TDLAS at 5739 cm^–1^, where the R(3) line of the first overtone band of HCl was probed, which was regarded as a feasible option considering the line intensity and equipment requirements. Different from the simulation using the HITRAN2016 and HITEMP2010 database, additional H_2_O line interfering the HCl absorption was observed at high temperatures, that is, above 1000 K. The absorption of the water line, if not properly accounted for, might introduce thousand ppm error to the HCl detection. Thus, a comprehensive investigation of the additional water line was conducted in the present work through the experimental measurement in homogeneous hot flue gas environments (1120–1950 K) provided by premixed laminar flames. Based on the measured temperature-dependent absorption spectra of H_2_O, the HCl absorption spectra can be well resolved after H_2_O absorption subtraction. The HCl concentration was obtained based on the fitting of the measured spectra using the HITRAN database. At high temperature, such as 1540 K, the detection limit of the present TDLAS system was estimated to be about 100 ppm⋅m. The detection limit strongly depends on temperature. Moreover, the technique has been applied to obtain the time resolved HCl release from burning PVC particles in a hot flue gas at 1790 K.

It is known that in combustion/gasification environments, water vapor is ubiquitous, and its spectral interference is a big challenge in species detection using IR-TDLAS. The present work provides important hot water spectra missing in the HITRAN and HITEMP databases. The experimental measurement of the hot water line absorption spectra in well-defined hot gas environments is necessary to improve the detection accuracy. The measurement process presented in this work is considered as a feasible routine that could be adopted in the development of IR-TDLAS techniques measuring various species in combustion/gasification environments.

## Supplemental Material

sj-xlsx-1-asp-10.1177_00037028211060866 – Supplemental Material for Quantitative Hydrogen Chloride Detection in Combustion Environments Using Tunable Diode Laser Absorption Spectroscopy with Comprehensive Investigation of Hot Water Interference Click here for additional data file. Supplemental Material, sj-xlsx-1-asp-10.1177_00037028211060866 for Quantitative Hydrogen Chloride Detection in Combustion Environments Using Tunable Diode Laser Absorption Spectroscopy with Comprehensive Investigation of Hot Water Interference by Wubin Weng, Jim Larsson, Joakim Bood, Marcus Aldén, and Zhongshan Li in Applied Spectroscopy
